# Supplementary material for: SYK kinase mediates brown fat differentiation and activation
Source: Nat Commun. 2017 Dec 13;8:2115. doi: 10.1038/s41467-017-02162-3 (PMC5727434; doi:10.1038/s41467-017-02162-3)
Supplement: Supplementary file 1 — Supplementary Information [file 41467_2017_2162_MOESM1_ESM.pdf]

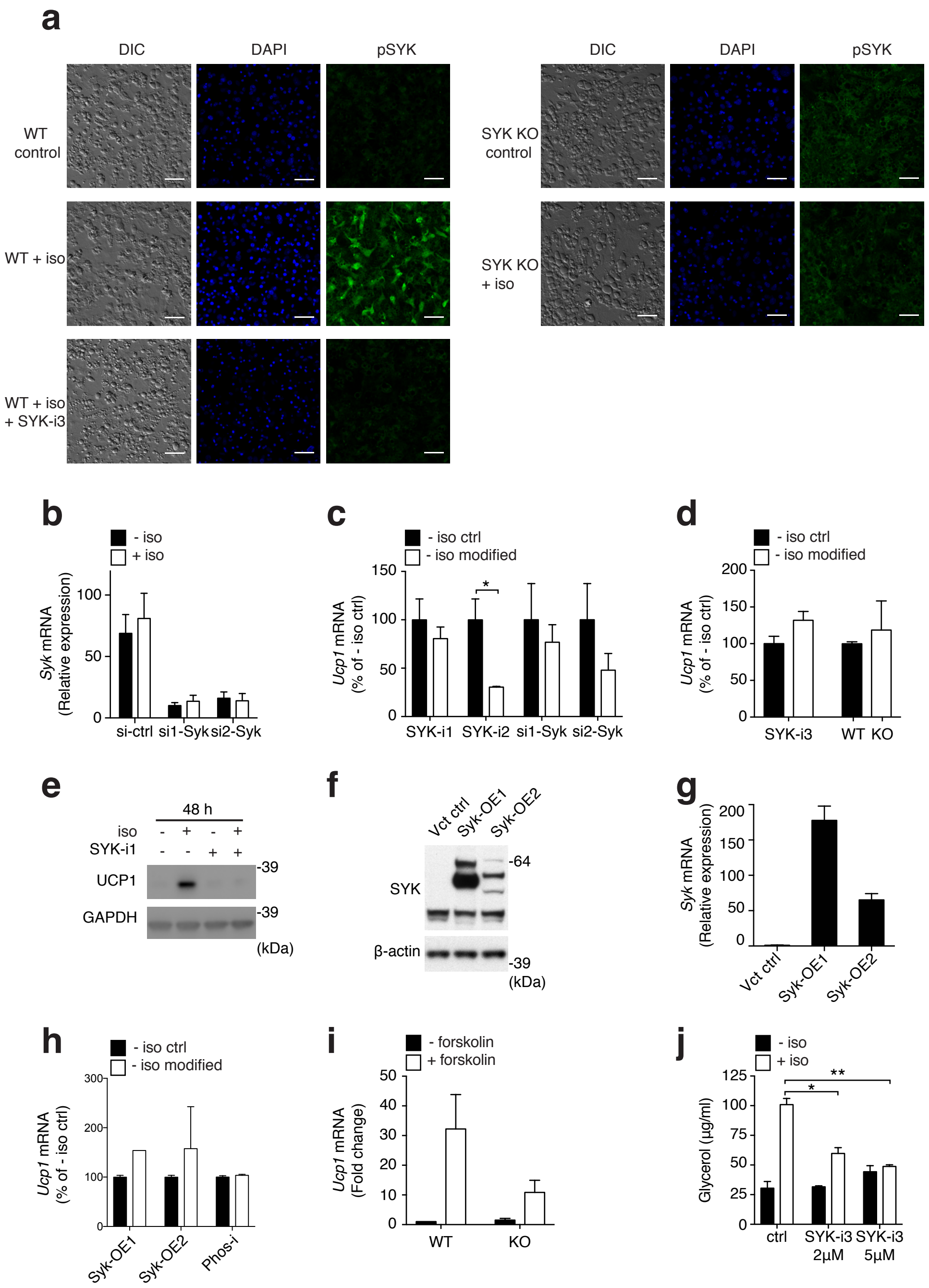

Supplementary Figure 1. SYK regulating *Ucp1* expression in response to β-adrenergic stimulation.

(a) Immunofluorescence of primary brown adipocytes stimulated with isoproterenol in the absence or presence of 5μM SYK-i3 (R406) and in primary day 8 brown adipocytes derived from CreERT2 Syk$^{flox/flox}$ mice (KO) treated with 4-hydroxy tamoxifen (4-OHT) for 9 days or WT controls, one representative IF of three independent replicates is shown. Scale bar = 50μM. **(b)** *Syk* RNA expression confirming knock down on day 10 of differentiation of immortalized brown pre-adipocytes following siRNA

mediated knock-down of *Syk* 4 days prior stimulated at day 10 of differentiation with isoproterenol for 6 h. **(c),** *Ucp1* RNA expression in the absence of isoproterenol for 6h of differentiated day 8 immortalized brown adipocytes pretreated with SYK-i1, SYK-i2 or following transfection with siRNA targeting *Syk* mRNA 4 days prior (si1-Syk and si2-Syk). **(d)** *Ucp1* RNA expression without isoproterenol in primary wild-type C57Bl/6 day 8 brown adipocytes *in vitro* pretreated with SYK-i3 or in primary day 8 brown adipocytes derived from CreERT2 $Syk^{flox/flox}$ mice (KO) or $Syk^{flox/flox}$ (WT) and both treated with 4-hydroxy tamoxifen (4-OHT) for 9 days. Mean % of iso stimulated control ± SEM of three independent experiments shown in **(c-d)**. **(e)** UCP1 protein expression in immortalized day8 differentiated brown adipocytes pretreated with 10μM Syk-i1 at day 8 of differentiation and then stimulated or not with isoproterenol for 48 h. **(f-g)** SYK protein **(f)** and *Syk* mRNA expression **(g)** following overexpression of wild-type mouse SYK in brown adipocytes (Syk-OE1 and Syk-OE2). (**h),** *Ucp1* RNA expression in primary day8 brown adipocytes after overexpression of wild-type mouse SYK (Syk-OE1 and Syk-OE2) or pretreatment with 1μM phosphatase inhibitor 3AC. Mean % of -iso control ± SEM of two independent experiments shown. **(i)**, Forskolin (1μM) induction of *Ucp1* expression in primary brown adipocytes derived from KO or WT and both treated with 4-hydroxy tamoxifen (4-OHT) for 9 days. Fold change of forskolin stimulated control ± SEM of three independent experiments. **j**, Cell culture medium glycerol content from primary white adipocytes at day8 treated with 1μM for 24h in the absence or presence of 2 or 5μM SYK-i3. For **(b-d, h, i)** two tailed unpaired Student's t-test was performed (*P < 0.05, **P < 0.005 and ***P < 0.0005). For **(j)** one way ANOVA on iso stimulated cells.

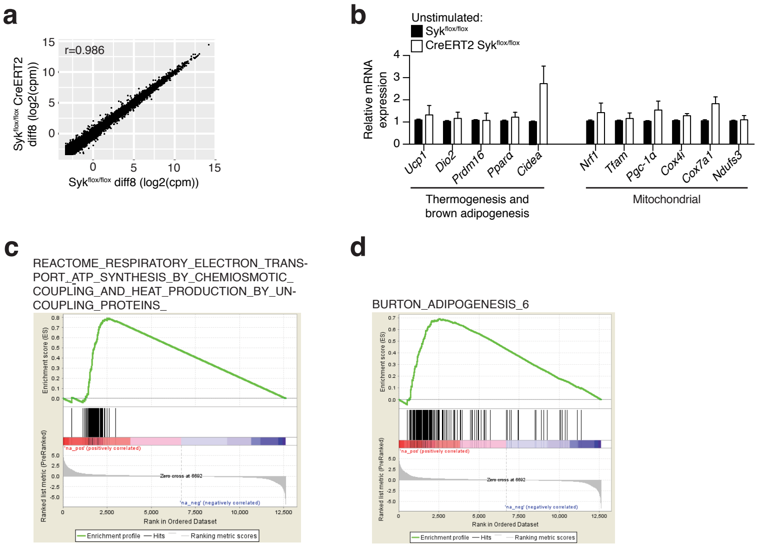

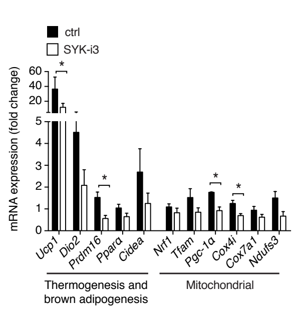

f

Target	Inhibitor name	*Ucp1* mRNA (% of + iso ctrl)
PI3K	PI 828<br>LY 294002	30,7<br>98,7
mTOR	KU0063794<br>Rapamycin	32,3<br>67,8
AKT	API-2<br>FPA 124	19,4<br>56,0
PKA	H89<br>KT 5720	51,6<br>66,2

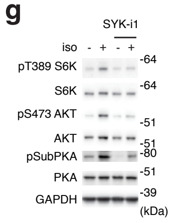

Supplementary Figure 2. SYK mediates expression of numerous brown fat characteristic genes in β-adrenergic activated brown adipocytes and signals the AKT/mTOR pathway to up-regulate *Ucp1*.

(a) RNAseq analysis of unstimulated day8 differentiated brown fat cells derived from CreERT2 Syk$^{flox/flox}$ (KO) mice and Syk$^{flox/flox}$ controls (WT) following continuous treatment with tamoxifen for 9 days. Values plotted are log2 counts per million reads (cpm) as estimated by edgeR from three independent replicates. **(b)** qPCR validation of the same cells as in **(a)** from three independent replicates. **(c-d)** Enrichment plots of the genes listed in the indicated gene set enrichment analyses (GSEA) for genes identified in the comparison of the fold change of stimulated and unstimulated WT controls versus stimulated and unstimulated KO brown adipocytes with a partial loss of SYK protein on day 8 of differentiation of three independent replicates. **(e)** Fold change of mRNA expression determined by qPCR in primary day8 differentiated brown adipocytes stimulated with isoproterenol for 6h and pre-treated or not with 2μM SYK-i3. Results shown were pooled from three independent experiments and shown as mean ±SEM. Asterisks denote statistical significance for individual genes (unpaired t-tests). **(f)** Table showing effect of PI3K, mTOR, AKT and PKA inhibitors on *Ucp1* expression from the screen as shown in **Figure 1a** from three independent replicates. Shown are mean % of isoproterenol stimulated control. **(g)** Immunoblotting of day8 immortalized brown adipocytes pretreated with 10μM SYK-i1 and stimulated or not with isoproterenol for 1h.

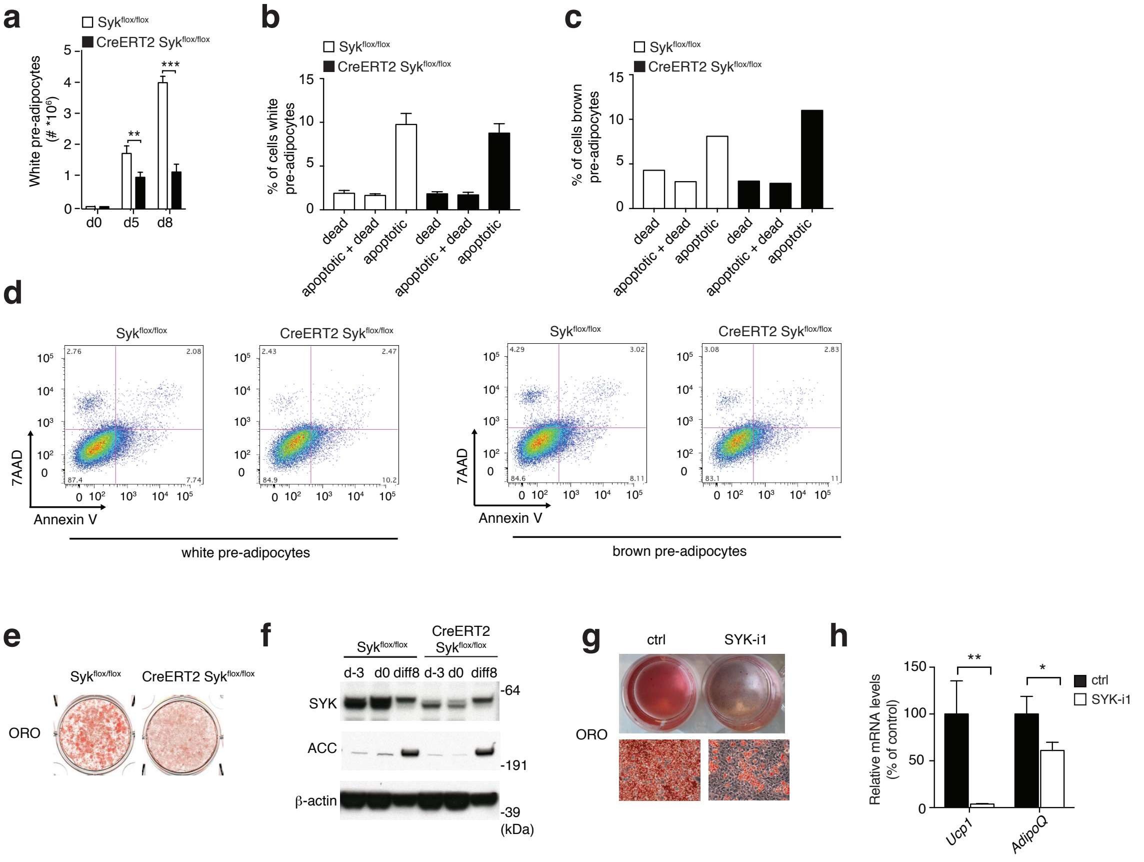

Supplementary Figure 3. SYK is essential for pre-adipocyte proliferation and for adipocyte differentiation.

(a) Cell numbers of white pre-adipocytes after seeding the CreERT2 Syk$^{flox/flox}$ (KO) and Syk$^{flox/flox}$ (WT) control pre-adipocytes at equal cell numbers (d0) following continuous 4-hydroxy tamoxifen (4-OHT) treatment beginning 3 days prior. Asterisks denote statistical significance ($p < 0.05$) across 3 independent experiments (two-tailed T-test). **(b-c)** Quantification of flow cytometry analysis of dead (7-AAD+), apoptotic and dead (7-AAD+, Annexin V+) or apoptotic cells (Annexin V+) of white **(b)** and brown pre-adipocytes derived from KO and WT mice **(c)**. **(d)** Representative flow cytometry plots used for quantification in (**b-c)** for white (left panel) and brown pre-adipocytes (right panel). **(e)** Representative Oil red oil staining (ORO) of brown pre-adipocytes isolated from KO and WT mice following 4-hydroxy tamoxifen (4-OHT) induced Cre mediated loss of SYK protein **(f)** in pre-adipocytes (d-3, d0) that were grown to confluence and induced to differentiate for another 8 days (diff8). Representative immunoblots of three independent replicates. (**g-h)** Oil red oil staining (ORO) **(g)** and mRNA expression **(h)** 10 days post induction of differentiation of immortalized brown pre-adipocytes in the presence of 2µM SYK-i1. For **(a, h)** two tailed unpaired Student's t-test was performed (*$P < 0.05$, **$P < 0.005$ and ***$P < 0.0005$).

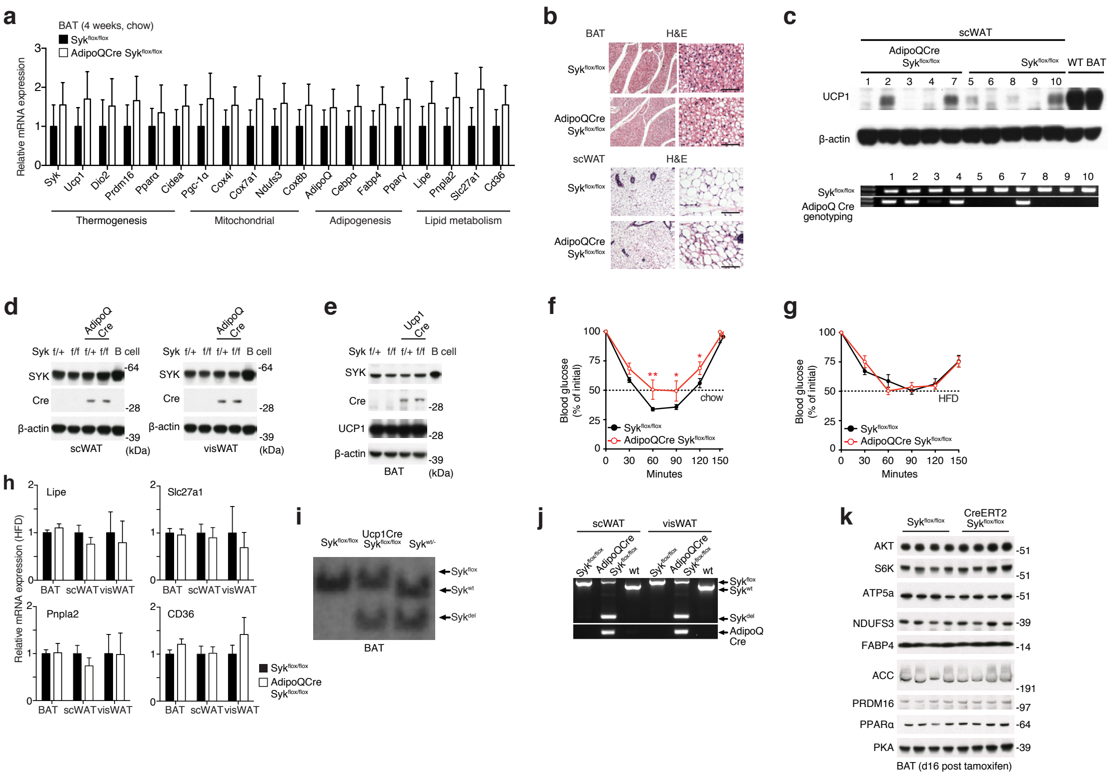

Supplementary Figure 4. ***Syk* deficiency is incompatible with brown fat formation and activation.** RNA expression of BAT depots **(a)** and histology of BAT and scWAT depots **(b)** from 4 week old AdipoQCre Syk$^{flox/flox}$ compared to Syk$^{flox/flox}$ littermate controls (n=5 each genotype) on a chow diet housed at 20°C. Scalebar **(b)** 100µm. **(c)** Immunoblotting of UCP1 and β-actin in scWAT of 8 week old AdipoQCre Syk$^{flox/flox}$ mice (n=5) or Syk$^{flox/flox}$ mice (n=5) littermates and wild-type BAT housed at 20°C. Samples were loaded according to genotyping (lower panel) for expression of AdipoQ Cre. **(d-e)** Representative immunoblots SYK and Cre from three independent isolations of scWAT and visWAT from 8 week old AdipoQCre Syk$^{flox/flox}$ mice, Syk$^{flox/flox}$ controls and heterozygous littermates **(d)** or of BAT from Ucp1Cre Syk$^{flox/flox}$ mice, Syk$^{flox/flox}$ controls and heterozygous littermates **(e)**. **(f-g)** Insulin tolerance test (ITT) of male AdipoQCre Syk$^{flox/flox}$ or Syk$^{flox/flox}$ control mice on a normal chow diet (chow) **(f)** or high fat diet (HFD) **(g)** for 11 weeks using the same mice as shown in **Figure 4e-f**.
RNA expression measured by qPCR of BAT, scWAT and visWAT from mice on a HFD for 25 weeks (n=6) **(h)**. Representative southern blot of BamHI digested genomic DNA of BAT from 6 week old mice with the indicated genotypes from two independent isolations **(i)** and PCR amplification of the deleted Syk^{flox} allele (top) and the AdipoQCre allele (bottom) in scWAT and visWAT of AdipoQCre Syk$^{flox/flox}$, Syk$^{flox/flox}$ mice, and wild-type mice with the indicated genotypes **(j)** of three independent isolations. **(k)** Representative immunoblots of two independent experiments of BAT from 10 week old CreERT2 Syk$^{flox/flox}$ (KO) mice and Syk$^{flox/flox}$ (WT) controls (n=4) 16 days after starting daily i.p. tamoxifen treatment for 5 consecutive days.

Figure 1b (red), Figure 1c (blue)

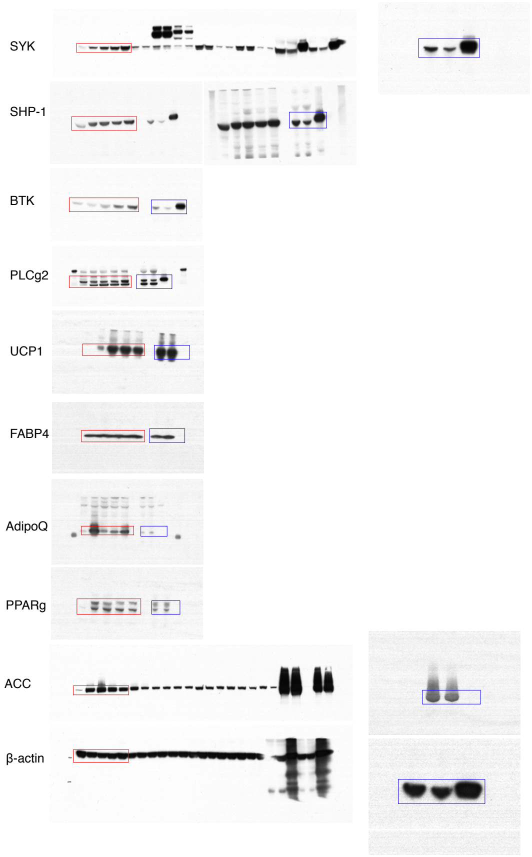

Supplementary figure 1e (green)

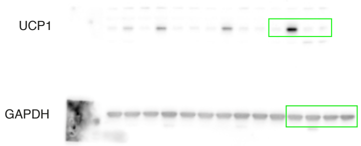

Supplementary figure 1f (orange)

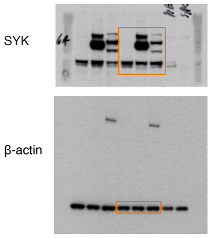

Supplementary Figure 5 related to Figure 1. Uncropped western blots related to Figure 1b (red boxes) and 1c (blue boxes). Uncropped western blots related to Supplementary Figure 1e (green boxes) and 1f (orange boxes).

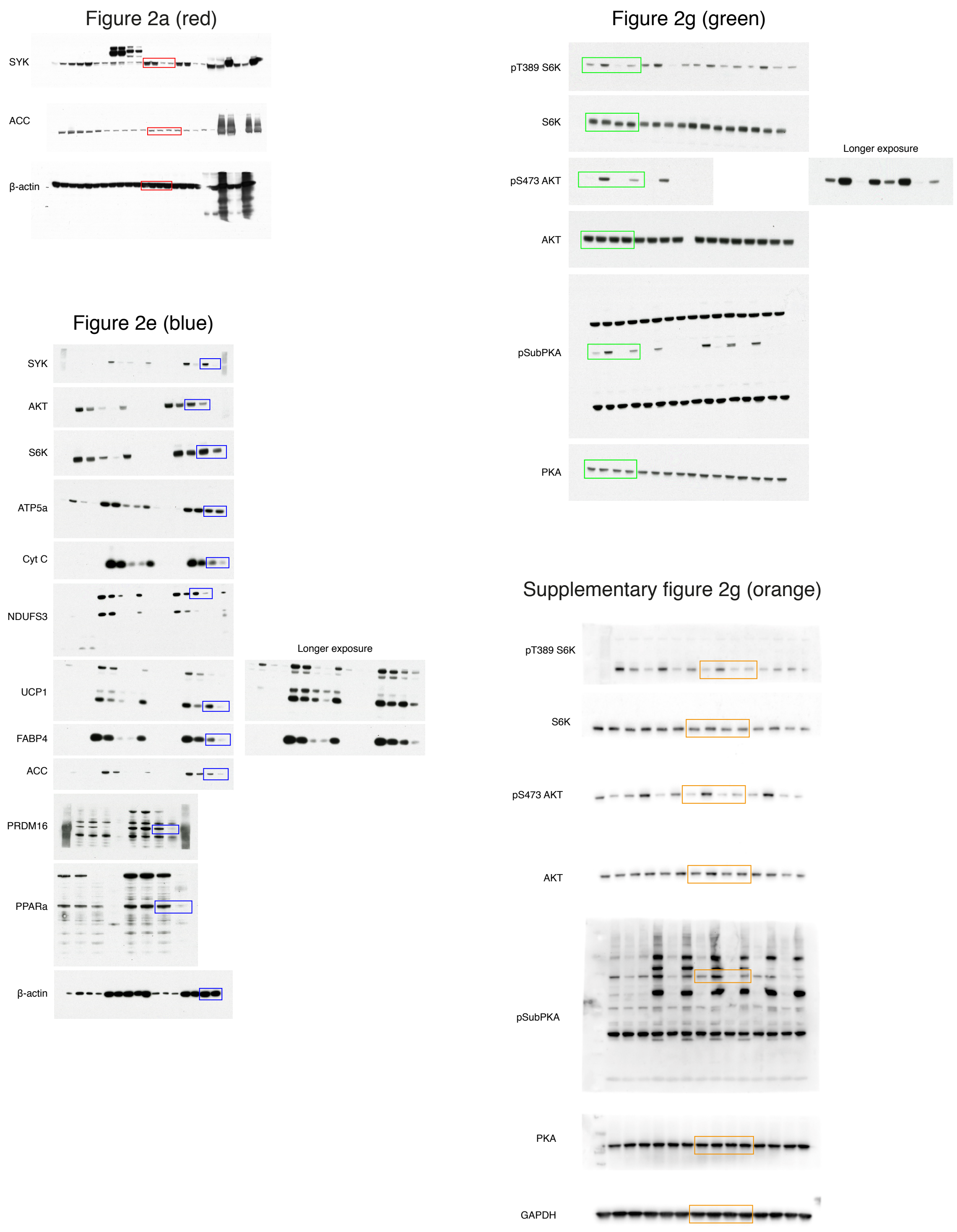

Supplementary Figure 6 related to Figure 2. Uncropped western blots related to Figure 2a (red boxes), 2e (blue boxes), 2g (green boxes) and Supplementary Figure 2g (orange boxes).

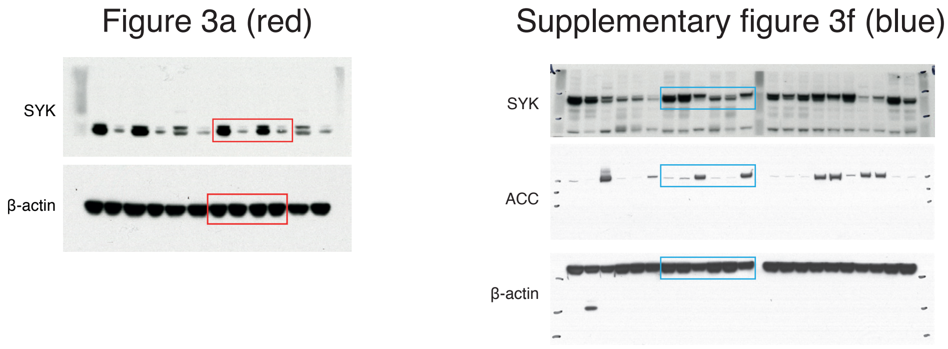

Supplementary Figure 7 related to Figure 3. Uncropped western blots related to Figure 3a (red boxes) and Supplementary 4f (blue boxes) and 4k (highlighted in green).

Figure 4d (red)

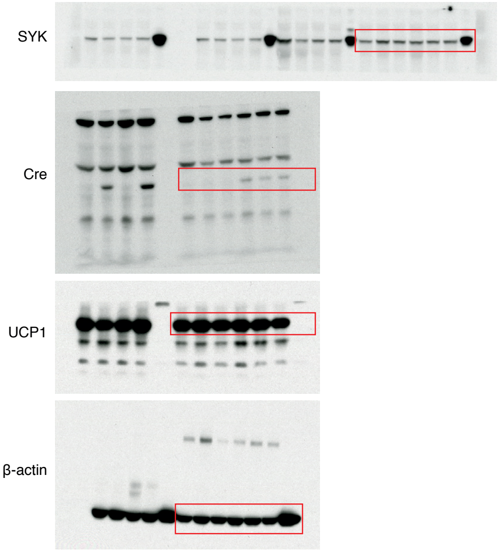

Figure 4g (blue)

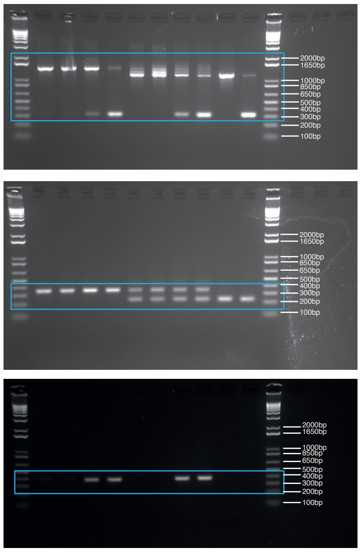

Figure 4h (green), Supplementary figure 4h (orange)

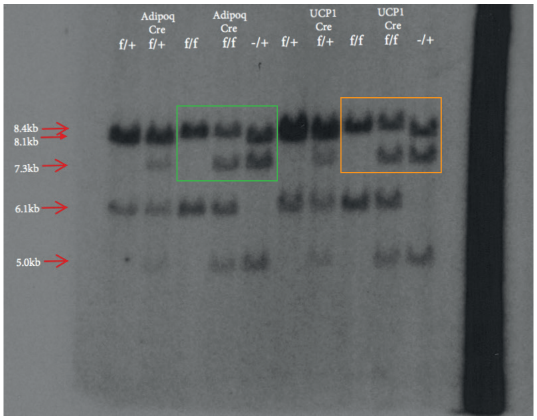

Figure 4k (pink)

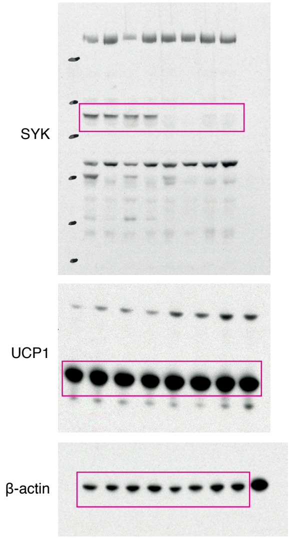

Supplementary Figure 8 related to Figure 4. Uncropped western blots related to Figure 4d (red boxes) and 4k (pink boxes). Uncropped agarose gels from PCR products related to Figure 4g (blue boxes) and uncropped blots from souther blots related to Figure 4h (orange boxes).

Supplementary figure 4c (red)

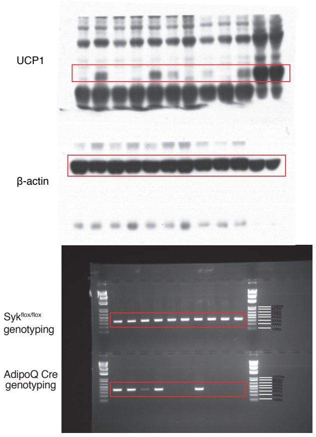

Supplementary figure 4d (blue)

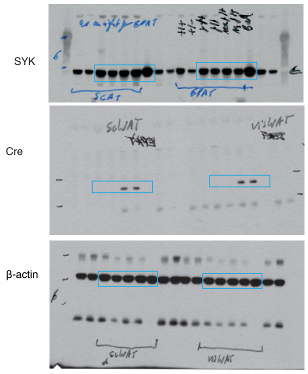

Supplementary figure 4e (green)

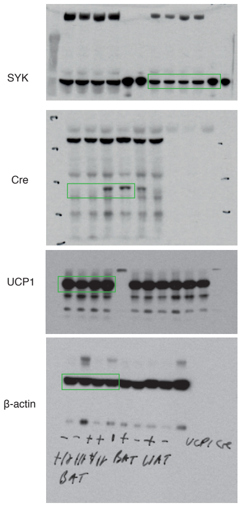

Supplementary figure 4i (pink)

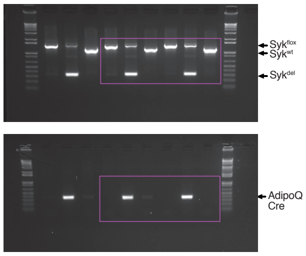

Supplementary figure 4j (orange)

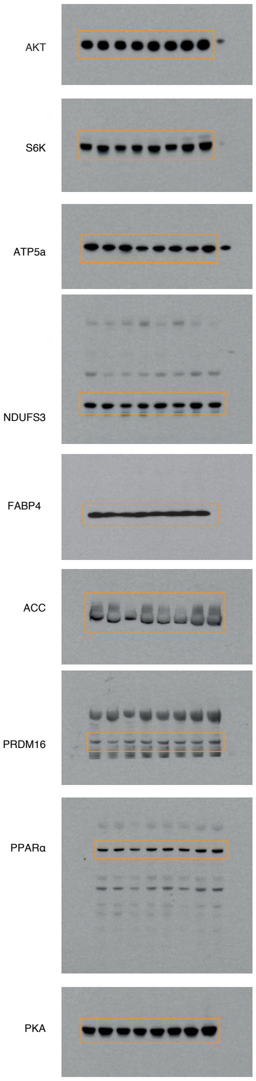

Supplementary Figure 9 related to Supplementary Figure 4. Uncropped western blots related to Supplementary Figure 4c (red boxes), 4d (blue boxes), 4e (green boxes) and 4j (orange boxes). Uncropped agarose gels from PCR products related to Supplementary Figure 4c (blue boxes) and 4i (pink boxes).

Supplementary Table 1. Library of kinase inhibitors used in the study

A complete list of kinase inhibitors including catalog number, product name and target kinase.

Cat. No.	Product name	Target kinase
414	AG 490	EGFR
431	ML 9 hydrochloride	MLCK
3439	NH 125	CaM Kinase III
541	Fasudil hydrochloride	ROCK
741	GF 109203X	PKC
1110	Genistein	EGFR
1130	LY 294002 hydrochloride	PI3K
1144	U0126	MEK
1213	PD 98059	MEK
1254	Y-27632 dihydrochloride	ROCK
1264	SB 202190	p38 MAPK
1284	Olomoucine	cdk
1300	LFM-A13	BTK
1321	ZM 336372	Raf
1366	ZM 449829	JAK3
1367	ZM 39923 hydrochloride	JAK3
1381	GW 5074	Raf
1397	PP 1	Src
1402	SB 203580 hydrochloride	p38 MAPK
1405	(-)-Terreic acid	BTK
1407	PP 2	Src
1459	SU 4312	VEGFR
1496	SP 600125	JNK
1580	Purvalanol A	cdk
1581	Purvalanol B	cdk
3544	KU 55933	ATM
1614	SB 431542	TGFbR1
1616	SB 216763	GSK-3
1617	SB 415286	GSK-3

Cat. No.	Product name	Target kinase
1777	Arctigenin	MEK
1937	NSC 693868	cdk
1962	SB 239063	p38 MAPK
1969	SL 327	MEK
2002	Ro 31-8220 mesylate	Broad Spectrum Inhibitor
2072	Aminopurvalanol A	cdk
2151	API-2	PKB
2238	GW 441756	TrkA
2239	GW 583340 dihydrochloride	EGFR
2272	Ro 08-2750	TrkA
2275	TBB	CK2
2291	1,2,3,4,5,6-Hexabromocyclohexane	JAK2
2415	HA 1100 hydrochloride	ROCK
2416	BIBX 1382 dihydrochloride	EGFR
2442	CGP 53353	PKC
2457	Arcyriaflavin A	cdk
2458	ZM 447439	Aurora
2471	ER 27319 maleate	Syk
2475	ZM 323881 hydrochloride	VEGFR
2499	ZM 306416 hydrochloride	VEGFR
2539	IKK 16	IKK
2542	Ki 8751	VEGFR
2558	10-DEBC hydrochloride	PKB
2559	TPCA-1	IKK
2560	SB 218078	Chk1
2591	TCS 359	FLT3
2605	PD 198306	MEK
2609	Ryuvidine	cdk
2611	IMD 0354	IKK
2639	CGK 733	ATR/ATM
2693	PHA 665752	cMET
2694	PD 407824	Chk1
2718	LY 364947	TGFbR1

Cat. No.	Product name	Target kinase
2731	CGP 57380	Mnk1
2768	PQ 401	IGF-1R
2814	PI 828	PI3K
2828	NU 7026	DNA-PK
2902	D 4476	CK1
2908	EO 1428	p38 MAPK
2910	H 89 dihydrochloride	PKA
2926	FPA 124	PKB
2977	GW 843682X	PLK
3000	Iressa	EGFR
3037	SU 5416	VEGFR
3063	1-Naphthyl PP1	Src
3572	GSK 650394	SGK
3194	BIO	GSK-3
3269	SD 208	TGFbR1
3271	Compound 401	DNA-PK
3314	BI 78D3	JNK
3318	SC 514	IKK
1289	KT 5823	PKG
1435	SQ 22536	Adenylyl cyclase
3093	Dorsomorphin dihydrochloride	AMPK
1292	Rapamycin	mTOR
1277	KN-62	CaMKII
1288	KT 5720	PKA
3824	KH7	Adenylyl cyclase
Merck millipore 570250	STO-609 acetate	CaMKK/AMPK
3725	KU0063794	mTOR
LC laboratories K-2152	K252a	Broad Spectrum Inhibitor

Supplementary Table 2. Genotyping primer and amplification product size

**Amplified PCR product**	**Forward Primer 5' - 3'**	**Reverse Primer 5' - 3'**
Syk deleted allele: 320 bp; *Syk* flox allele: 1428 bp; *Syk* wt allele: 1186 bp	GCCCGTTCTGTGCCTACTGG	GCTGGTTCCCTTTTCCTTCC
Syk flox allele: 349 bp; *Syk* wt allele: 234 bp	GCCCGTTCTGTGCCTACTGG	TAGCTAACCAAACCCACGGC
AdipoQCre: around 373 bp	CATGATGCAGGTCCTGATTG	ATGTTTAGCTGGCCCAAATG
Ucp1Cre: around 654 bp	TGTCCGTTTGCCGGTCGTGG	ATCCCTTCCAGGGCGCGAGT
CreERT2: around 689 bp	ATACCGGAGATCATGCAAGC	TCCAGAGACTTCAGGGTGCT

Supplementary Table 3. Mouse specific primer pairs used for Quantitative Real-time PCR

Gene name	Forward Primer 5' - 3'	Reverse Primer 5' - 3'
18S	GTAACCCGTTGAACCCCATT	CCATCCAATCGGTAGTAGC
18S	ACCGCAGCTAGGAATAATGGA	GCCTCAGTTCCGAAAACCA
Tbp	ACCCTTCACCAATGACTCCTATG	ATGATGACTGCAGCAAATCGC
Cebpa	TGCGCAAGAGCCGAGATAAA	CCTTCTGTTGCGTCTCCACG
Pparγ	GTGCCAGTTTCGATCCGTAGA	GGCCAGCATCGTGTAGATGA
Cidea	TGCTCTTCTGTATCGCCCAGT	GCCGTGTTAAGGAATCTGCTG
Dio2	CAGTGTGGTGCACGTCTCCAATC	TGAACCAAAGTTGACCACCAG
Pgc-1α	CCCTGCCATTGTTAAGACC	TGCTGCTGTTCCTGTTTTC
Pparα	AGAGCCCCATCTGTCCTCTC	ACTGGTAGTCTGCAAAACCAAA
Prdm16	CAGCACGGTGAAGCCATTC	GCGTGCATCCGCTTGTG
Ucp1	ACTGCCACACCTCCAGTCATT	CTTTGCCTCACTCAGGATTGG
Ucp1	GGCATTCAGAGGCAAATCAGCT	CAATGAACACTGCCACACCTC
Cox8b	GAACCATGAAGCCAACGACT	GCGAAGTTCACAGTGGTTCC
Cox4i	ACCAAGCGAATGCTGGACAT	GGCGGAGAAGCCCTGAA
Cox7a1	CAGCGTCATGGTCCAGTCTGT	AGAAAACCGTGTGGCAGAGA
Tfam	GCAGCTAACTCCAAGTCAGC	CCGAATCATCCTTTGCCTCC
Nrf1	GTTGGATGAGTACACGACGC	GAATTAACCTCCTGTGGCGC
Ndufs3	CAGGGATCACACCAATGCAC	AGTCAATGGGTGTCAGCTCA
Syk	GGAAGAGAGCAACTTTGTGC	GTCTGGGCCTTGTAGTAGTT
AdipoQ	CGATTGTCAGTGGATCTGACG	CAACAGTAGCATCCTGAGCCCT
Fabp4	ACAAGCTGGTGGTGGAATGTG	CCTTTGGCTCATGCCCTTT
Lipe	CCGCTGACTTCCTGCAAGAG	CTGGGTCTATGGCGAATCGG

**Gene name**	**Forward Primer 5' - 3'**	**Reverse Primer 5' - 3'**
Pnpla2	GGTGACCATCTGCCTTCCAG	TGCAGAAGAGACCCAGCAGT
Slc27a1	CCGTATCCTCACGCATGTGT	CTCCATCGTGTCCTCATTGAC
Cd36	TCTGTTGGAACAGAGGATGA	TGGAACCAAACTGAGGAATG
Syk	GAGTCCTGGATGCTGGTGAT	ACTTCATCCCCATGGAAACC
Glut4	CTGTCGCTGGTTTCTCCAACT	CCCATAGCATCCGCAACATA

Supplementary Table 4. Antibodies

The following antibodies were used in immunoblotting (WB) or Immunfluorescence (IF) experiments.

Target antigen	Clone / Catalog #	Manufacturer	Application
SYK	D3Z1E (rabbit monoclonal)	CST	WB (1:1,000)
pSYK	sc-293118 (rabbit polyclonal)	SCBT	IF-P (1:50)
SHP1	Y476 (rabbit monoclonal)	Abcam	WB (1:1,000)
PLCγ2	Q-20 (sc-407)	SCBT	WB (1:1,000)
BTK	D3H5 (rabbit monoclonal)	CST	WB (1:1,000)
pS473 AKT	D9E (rabbit monoclonal) and #4058	CST	WB (1:1,000)
AKT	# 9272 (rabbit polyclonal)	CST	WB (1:1,000)
pT389 S6K	#9234 (Rabbit monoclonal)	CST	WB (1:1000)
S6K	#2708 (Rabbit monoclonal)	CST	WB (1:1000)
GAPDH	8245 (mouse monoclonal)	Abcam	WB (1:20.000)
Cytochrome C	37BA11/ab110325 (mouse monoclonal)	Abcam	WB (1:10,000)
ATP5a	15H4C4 (mouse monoclonal)	Abcam	WB (1:1,000)
NDUFS3	3F9DD2 (mouse monoclonal)	Abcam	WB (1:1000)
UCP1	Ab10983 (rabbit polyclonal)	Abcam	WB (1:1,000)
ACC	Ab109368 (rabbit monoclonal)	Abcam	WB (1:1,000)
Anti-rabbit IgG	Anti-rabbit IgG, HRP linked (#7074)	CST	WB (1:5,000)
Anti-mouse IgG	Anti-mouse IgG, HRP linked (#7076)	CST	WB (1:5,000)
Phospho-(Ser/Thr) PKA Substrate	#9621 (Rabbit polyclonal)	CST	WB (1:1000)
PKA C-a	#4782 (Rabbit	CST	WB (1:1000)
β-actin	A5441 (mouse monoclonal)	Sigma-Aldrich	WB (1:10,000)
PPARα	H98 (rabbit monoclonal)	SCBT	WB (1:1,000)
PRDM16	ab118573 (rabbit polyclonal)	Abcam	WB (1:1,000)

Target antigen	Clone / Catalog #	Manufacturer	Application
FABP4	# 2120 (rabbit polyclonal)	CST	WB (1:1,000)
Cre	#15036 (rabbit monoclonal)	CST	WB (1:1,000)
