# Supplementary material for: SYK kinase mediates brown fat differentiation and activation
Source: Nat Commun. 2017 Dec 13;8:2115. doi: 10.1038/s41467-017-02162-3 (PMC5727434; doi:10.1038/s41467-017-02162-3)
Supplement: Supplementary file 3 — Description of Additional Supplementary Files [file 41467_2017_2162_MOESM3_ESM.docx]

**Description of Additional Supplementary Files**

File Name: Supplementary Data 1

Description: Transcriptional SYK targets are enriched for pathways required for brown fat differentiation. Enrichment analysis of SYK transcriptional targets determined by mRNA sequencing of isoproterenol stimulated day 8 brown adipocytes derived from CreERT2 Sykflox/flox mice treated with tamoxifen in vitro.
